# Choice of postoperative radiation for stage IIIA pathologic N2 non-small cell lung cancer: impact of metastatic lymph node number

**DOI:** 10.1186/s13014-017-0946-1

**Published:** 2017-12-29

**Authors:** Siwei Wang, Zhifei Ma, Xiangbao Yang, Yajing Wang, Youtao Xu, Wenjia Xia, Rui Chen, Mantang Qiu, Feng Jiang, Rong Yin, Lin Xu, Keping Xu

**Affiliations:** 1 Department of Thoracic Surgery, Jiangsu Cancer Hospital & Jiangsu Institute of Cancer Research & Nanjing Medical University Affiliated Cancer Hospital, Jiangsu Key Laboratory of Molecular and Translational Cancer Research, Nanjing, 210009 China; 2 The Fourth Clinical College of Nanjing Medical University, Nanjing, 210000 China; 3 Department of Thoracic Surgery, Huai’an First People’s Hospital, Nanjing Medical University, Huai’an, 223300 China; 4 Department of Cardiothoracic Surgery, Taixing People’s Hospital, The Affiliated Taixing Hospital of Yangzhou University, Taixing, 225400 China

**Keywords:** Postoperative radiotherapy (PORT), Non-small cell lung cancer (NSCLC), Lymph nodes (LNs)

## Abstract

**Background:**

Postoperative radiation (PORT) is an option for non-small cell lung cancer (NSCLC) patients with resectable stage IIIA pathological N2 status (pN2). For patients with PORT, this study aims to investigate the impact of the exact number of positive lymph nodes (LNs) on overall survival (OS) and lung cancer-specific survival (LCSS).

**Methods:**

Within the Surveillance, Epidemiology, and End Results database, we identified 3373 patients with stage IIIA pathological N2 status (pN2) NSCLC who underwent a lobectomy or pneumonectomy from 2004 to 2013. OS and LCSS were compared among patients coded as receiving PORT or observation. The proportional hazards model was applied for investigation.

**Results:**

OS and LCSS favored PORT for patients with stage IIIA (pN2) NSCLC. Multivariable analyses showed that PORT and the exact number of positive LNs (*n* ≤ 3) were independently associated with better OS and LCSS. Both better OS and LCSS emerged for positive LNs (*n* > 3) after the use of PORT in survival analyses, whereas the benefits of OS and LCSS were not observed anymore for positive LNs (*n* ≤ 3) group. More importantly, multivariable analyses showed that the use of PORT is an independent risk factor of survival for positive LNs (n > 3) but not for positive LNs (n ≤ 3).

**Conclusions:**

In Stage IIIA (pN2) NSCLC, the use of PORT demonstrated better survival results than no PORT for patients with positive LNs (n > 3), but not for patients with positive LNs (n ≤ 3).

**Electronic supplementary material:**

The online version of this article (10.1186/s13014-017-0946-1) contains supplementary material, which is available to authorized users.

## Background

The presence of histologically confirmed lymph node metastases is an important prognostic factor for many malignancies. In non-small cell lung cancer (NSCLC), the nodal status with metastases has been suggested to be of significance. For patients with pathologic N2 NSCLC that is considered resectable, complete surgical resection is a favor choice of the management of localized non-small-cell cancer and the use of adjuvant platinum-based chemotherapy is also considered the standard of care presently [1, 2].

However, even after complete surgical resection and adjuvant chemotherapy, node-positive patients still have a 20% to 40% risk of localregional recurrence (LRR), and LRR correlates independently with worse OS for patients with NSCLC [2, 3]. Thus, postoperative radiation therapy (PORT) is often recommended to improve local tumor control and survival in IIIA (N2) NSCLC patients with good performance status. Additionally, for resectable stage IIIA N2 patients, National Comprehensive Cancer Network (NCCN) guidelines (Version4. 2016) also support the use of PORT (sequential or concurrent chemoradiation) for N2 nodal status patients regardless of whether the surgical margins are positive. Two single-center retrospective studies and one previous SEER based study suggested that the use of PORT improved survival for patients with N2 nodal disease [4–6]. Another postoperative trial also demonstrated a benefit to PORT in N2 disease [7]. However, a previous meta-analysis of randomized trials demonstrated no benefit with PORT, and the use of PORT could even result in a decrease in OS due to the cardiac and pulmonary toxicity from the radiotherapy itself [8]. In addition, a recent randomized trial refer to whether PORT is benefit or not for N2 NSCLC patients (NCT00410683, Lung ART in Paris) is still recruiting patients, which indicates that PORT or not was still a controversial issue.

In NSCLC, the number of nodal stations with metastases has been previously demonstrated to have significances on the survival of N2 diseases with PORT [9, 10]; however, few studies focused on the numbers of positive LNs, and so far only one single-center study demonstrated the total number of positive LNs seems to be an independent prognostic indicator in patients with pN2 NSCLC [11]. Therefore, it is valuable to further analyze whether the number of positive LNs could impact the prognosis in pN2 patients with PORT.

## Methods

### Data collection

**Fig. 1:**
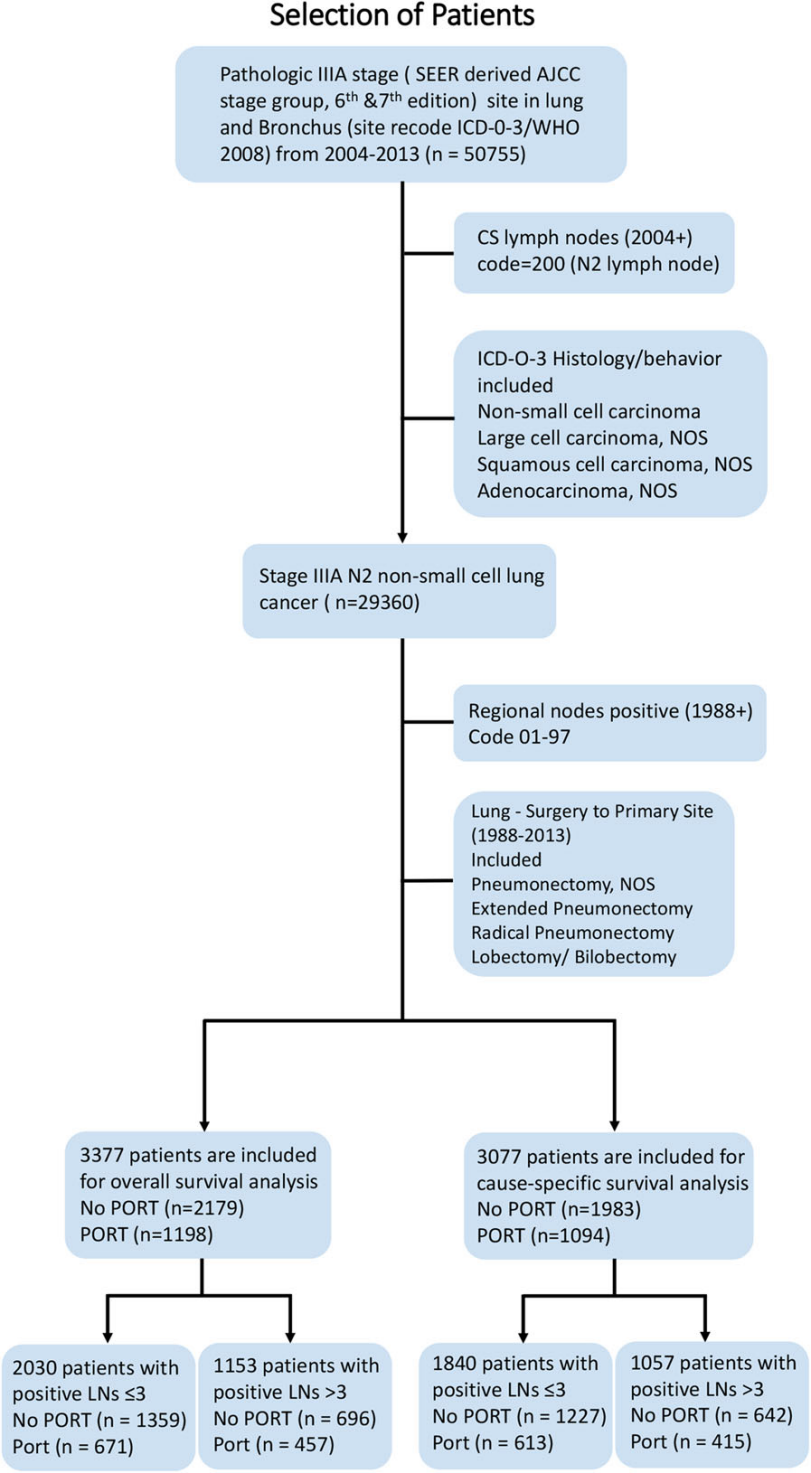
Selection of patients. Abbreviations: SEER, Surveillance, Epidemiology, and End Results database; AJCC, American Joint Committee on Cancer; CS, Collaborative Stage Data Collection System; ICD-O-3, International Classification of Diseases for Oncology, 3rd Edition; WHO, World Health Organization; NOS, Not Otherwise Specified (a CS defined method); LNs, lymph nodes

The Surveillance, Epidemiology, and End Results (SEER) database is a national cancer surveillance program that collects information on all incident cancer cases from 18 areas of United States and covers approximately 26% of the population. In this study, identified data for patients with stage IIIA pathological N2 NSCLC were obtained from the SEER database for patients treated from January 2004 to December 2013. Pathologic IIIA stage patients derived from AJCC stage group (6th and 7th edition). N2 LNs status were defined according to CS LNs codes manual. The specific histologic types selected were those coded as non-small cell carcinoma, large cell carcinoma NOS, adenocarcinoma NOS and squamous cell carcinoma NOS. We then only chose patients with positive LNs according to regional nodes positive codes. Patients were finally included if they underwent a radical surgery of either a lobectomy or pneumonectomy. Subsequently, only those patients coded as receiving no radiation and/or cancer-directed surgery were considered not treated by postoperative radiotherapy, and those who coded as radiation after surgery were defined treated by postoperative radiotherapy. Radiation method was then restricted to beam radiation and radiation NOS according to radiation codes. Overall survival (OS) and lung cancer-specific survival (LCSS) were determined from SEER cause-specific death classification and SEER other cause of death classification codes. OS was defined as the time from surgery until death as a result of any cause, and LCSS was defined as the interval from surgery until death as a result of lung cancer. To reduce the immortal time bias, we excluded patients who survived less than 4 months. Fig. 1 details the selection process for inclusion of patients.

Categoric variables included patient age at diagnosis (<65 v. ≥65 years), sex, race, location (main bronchus, upper lobe, middle lobe, lower lobe, overlapping and unspecified), tumor size (≤3·0, 3·1 to 5·0, 5.1 to 7·0, ≥7·0 cm and unknown), T stage (T1, T2, T3 and Tx), laterality (right, left and unspecified), histology (non-small cell carcinoma, adenocarcinoma NOS, squamous cell carcinoma, NOS and large cell carcinoma NOS), surgery type (lobectomy and pneumonectomy), number of positive LNs in classification one (1, 2, 3, 4, 5, 6, 7, ≥8 and number unspecified), and classification two (≤ 3 v. >3, number unspecified excluded). Information on margin status, use of adjuvant chemotherapy, and specific radiotherapy technique (dose, beam energy, and so on) was not available with the SEER database and is no included in the analysis. Patients were divided into no postoperative radiotherapy (PORT) and postoperative radiotherapy groups according to whether they underwent postoperative radiotherapy.

### Statistical analysis

The Pearson χ2 test was used to analyze categoric variables. We used Kaplan-Meier method to determine OS and LCSS for patients underwent PORT or not. The log-rank test was used to compare the survival curves between Port and No PORT groups. Multivariable Cox proportional hazards models were used to calculate adjusted hazard ratios (HRs) and their 95% CIs relating to the variables as described. Results were considered to be statistically significant when *P* < 0.05. All data were analyzed using the SPSS 22.0 (SPSS, Chicago, IL), and the survival curve was drawn with GraphPad Prism 5.0 (GraphPad Software, San Diego, CA).

## Results

### Baseline characteristics and outcomes

A total of 3377 stage IIIA (N2) NSCLC patients with positive LNs were included in overall survival analysis, and 3077 patients were included in lung cancer-specific survival analysis. In the OS analysis, comparative treatment strategy was PORT in 1198 patients (35·5%) and no PORT in 2179 (64·5%). In the LCSS analysis, 1094 patients (35·6%) and 1983 (64·4%) underwent PORT and no PORT separately. Table 1 details the baseline characteristics. According to the results illustrated above, PORT was less performed in stage IIIA (N2) NSCLC patients, especially in elderly patients. And patients underwent lobectomy constitute the vast majority of included patients. Additionally, no significance differences emerged in sex, race location, tumor size, T stages, laterality and histology between PORT and No PORT patients.

**Table 1:** Baseline Characteristics of Patients with stage IIIA pN2 status NSCLC

	Baseline Characteristics of Patients in OS analysis			Baseline Characteristics of Patients in LCSS analysis		
	No.(%) of Patients (*n* = 3377)			No.(%) of Patients (*n* = 3077)		
Demographic	No Port (*n* = 2179)	Port (*n* = 1198)	*P* Value for χ2	No Port (*n* = 1983)	Port (*n* = 1094)	*P* Value for χ2
Age at diagnosis, years						
< 65	887 (40.7%)	619 (51.7%)	<0.001	829(41.8%)	573(52.4%)	<0.001
≥ 65	1292 (59.3%)	579 (48.3%)		1154(58.2%)	521(47.6%)	
Sex						
Male	1108 (50.4%)	605 (50.5%)	0.846	996(50.2%)	539(49.3%)	0.611
Female	1071 (49.6%)	593 (49.5%)		987(49.8%)	555(50.7%)	
Race						
White	1789 (82.1%)	967 (80.7%)	0.564	1624(81.9%)	882(80.6%)	0.622
Black	209 (9.6%)	120 (10.0%)		193(9.7%)	110(10.1%)	
Other	181 (8.3%)	111 (9.3%)		166(8.4%)	102(9.3%)	
Location						
Main bronchus	28 (1.3%)	14 (1.2%)	0.106	27(1.4%)	11(1.0%)	0.186
Upper lobe	1221 (56.0%)	728 (60.8%)		1116(56.3%)	663(60.6%)	
Middle lobe	102 (4.7%)	56 (4.7%)		93(4.7%)	52(4.8%)	
Lower lobe	760 (34.9%)	366 (30.5%)		683(34.4%)	336(30.7%)	
Overlapping/lung, NOS	68 (3.1%)	34 (2.8%)		64(3.2%)	32(2.9%)	
Tumor size, cm						
≤ 3.0	939 (43.1%)	507 (42.3%)	0.805	849(42.8%)	464(42.4%)	0.73
3.1 to 5.0	711 (32.6%)	402 (33.6%)		645(32.5%)	370(33.8%)	
5.1 to 7.0	323 (14.8%)	168 (14.0%)		301(15.2%)	153(14.0%)	
≥ 7.1	161 (7.4%)	99 (8.3%)		144(7.3%)	87(8.0%)	
Unknown	45 (2.1%)	22 (1.8%)		44(2.2%)	20(1.8%)	
T stage						
T1	611 (28.0%)	326 (27.2%)	0.155	550(27.7%)	299(27.3%)	0.355
T2	1305 (59.9%)	701 (58.5%)		1181(59.6%)	638(58.3%)	
T3	244 (11.2%)	164 (13.7%)		233(11.7%)	150(13.7%)	
TX	19 (0.9%)	7 (0.6%)		19(1.0%)	7(0.7%)	
Laterality						
Right	1206 (55.3%)	687 (57.4%)	0.241	1101(55.5%)	640(58.6%)	0.098
Left	973 (44.7%)	509 (42.6%)		882(44.5%)	452(41.4%)	
Unspecified	/	2		/	2	
Histology						
Non-small cell carcinoma	141 (6.5%)	81 (6.8%)	0.113	128(6.5%)	71(6.5%)	0.076
Adenocarcinoma, NOS	1366 (62.7%)	795 (66.4%)		1251(63.1%)	738(67.4%)	
Squamous cell carcinoma, NOS	609 (27.9&)	294 (24.5%)		546(27.5%)	259(23.7%)	
Large cell carcinoma, NOS	63 (2.9%)	28 (2.3%)		58(2.9%)	26(2.4%)	
Surgery type						
Lobectomy	1894 (86.9%)	1087 (90.7%)	0.001	1719(86.7%)	999(91.3%)	<0.001
Pneumonectomy	285 (13.1%)	111 (9.3%)		264(13.3%)	95(8.7%)	
Number of positive lymph nodes						
1	655 (30.1%)	286(23.9%)	<0.001	588(29.7%)	263(24.1%)	0.009
2	426 (19.6%)	212(17.7%)		386(19.5%)	194(17.7%)	
3	278 (12.8%)	173(14.4%)		253(12.8%)	156(14.3%)	
4	198 (9.1%)	116(9.7%)		183(9.2%)	103(9.4%)	
5	135 (6.2%)	103(8.6%)		124(6.3%)	96(8.8%)	
6	101 (4.6%)	59(4.9%)		92(4.6%)	54(4.9%)	
7	62(2.8%)	41(3.4%)		56(2.8%)	35(3.2%)	
≥ 8	200(9.2%)	138(11.5%)		187(9.4%)	127(11.6%)	
No. of unspecified	124(5.9%)	70(5.9%)		114(5.7%)	66(6.0%)	
Number of positive lymph nodes						
≤ 3	1359 (66.1%)	671 (59.5%)	<0.001	1227 (65.7%)	613 (59.6%)	0.001
> 3 (Unspecified Excluded)	696 (33.9%)	457 (40.5%)		642 (34.3%)	415 (40.4%)	

*Abbreviations*: *NSCLC* non-small cell lung cancer, *OS* overall survival, *LCSS* lung cancer-specific survival, *NOS* not otherwise specified

**Fig. 2:**
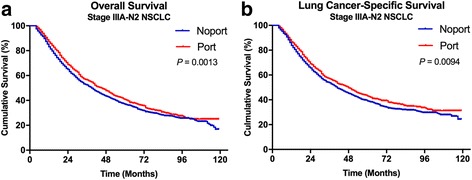
Overall and lung cancer-specific survivals in patients with stage IIIA pN2 status non-small cell lung cancer (NSCLC) undergoing No PORT or PORT (**a** and **b**). Abbreviations: PORT, Postoperative radiation. NSCLC, Non-small cell lung cancer

The survival analysis by Kaplan-Meier plots showed that PORT was significantly associated with better OS (log-rank test, *p* = 0·0013) and LCSS (log-rank test, *p* = 0·0094) for all NSCLC patients (Fig. 2a and b). These results were similar to E, Lally’s study [4].

The Cox proportional hazard regression analyses (Additional file 1: Table S1) then demonstrated that older age, T2 stage, T3 stage, 4 positive LNs, 5 positive LNs, 6 positive LNs, 7 positive LNs and ≥8 positive LNs had negative impacts on survival. Additionally, results showed that patients benefited from PORT significantly (OS: HR = 0·854, 95% CI, 0·76 to 0·941, *P* = 0·001; LCSS: HR = 0·855, 95%CI, 0·769 to 0·95, P = 0·004).

Based on the preliminary analysis shown above, we combined the number of positive LNs categories (1, 2, 3, 4, 5, 6, 7, ≥8 and number unspecified) into three kinds (≤3, >3 and number unspecified). The Cox proportional hazard regression analyses (Additional file 1: Table S2) demonstrated that older age (OS: HR = 1·407; 95% CI, 1·281 to 1·545; *P* < 0·001; LCSS: HR = 1·428; 95% CI, 1·290 to 1·581; P < 0·001), T2 stage (OS: HR = 1·254; 95% CI, 1·082 to 1·453; *P* = 0·003; LCSS: HR = 1·247; 95% CI, 1·061 to 1·466; P = 0·007) and T3 stage (OS: HR = 1·770; 95% CI, 1·461 to 2·144; P < 0·001; LCSS: HR = 1·810; 95% CI, 1·472 to 2·225; P < 0·001) had a negative impact on survival. Notably, positive LNs (*n* > 3) was found independently associated with poorer survival (OS: HR = 1·379, 95% CI, 1·253 to 1·519, P < 0·001; LCSS: HR = 1·415, 95% CI, 1·274 to 1·571, P < 0·001). In addition, patients still benefited from the use of PORT (OS: HR = 0·860, 95% CI, 0·781 to 0·947, P = 0·002; LCSS: HR = 0·862, 95% CI, 0·775 to 0·957, P = 0·006) significantly.

### Comparison of positive lymph nodes (*n* ≤ 3 and *n* > 3)

Subset characteristic analyses were then performed for patients classified by the number of positive LNs (n ≤ 3 and *n* > 3). Patients with unspecified number of positive LNs were excluded, and so 194 and 180 patients were excluded from the following OS and LCSS multivariable analyses. Additional file 1: Table S3 and Table S4 detailed the baseline characteristics of OS and LCSS. No significance differences emerged in sex, race location, tumor size, T stages, laterality and histology between two groups, except T stage categories in the No. ≤3 group of LCSS patients’ characteristic.

**Fig. 3:**
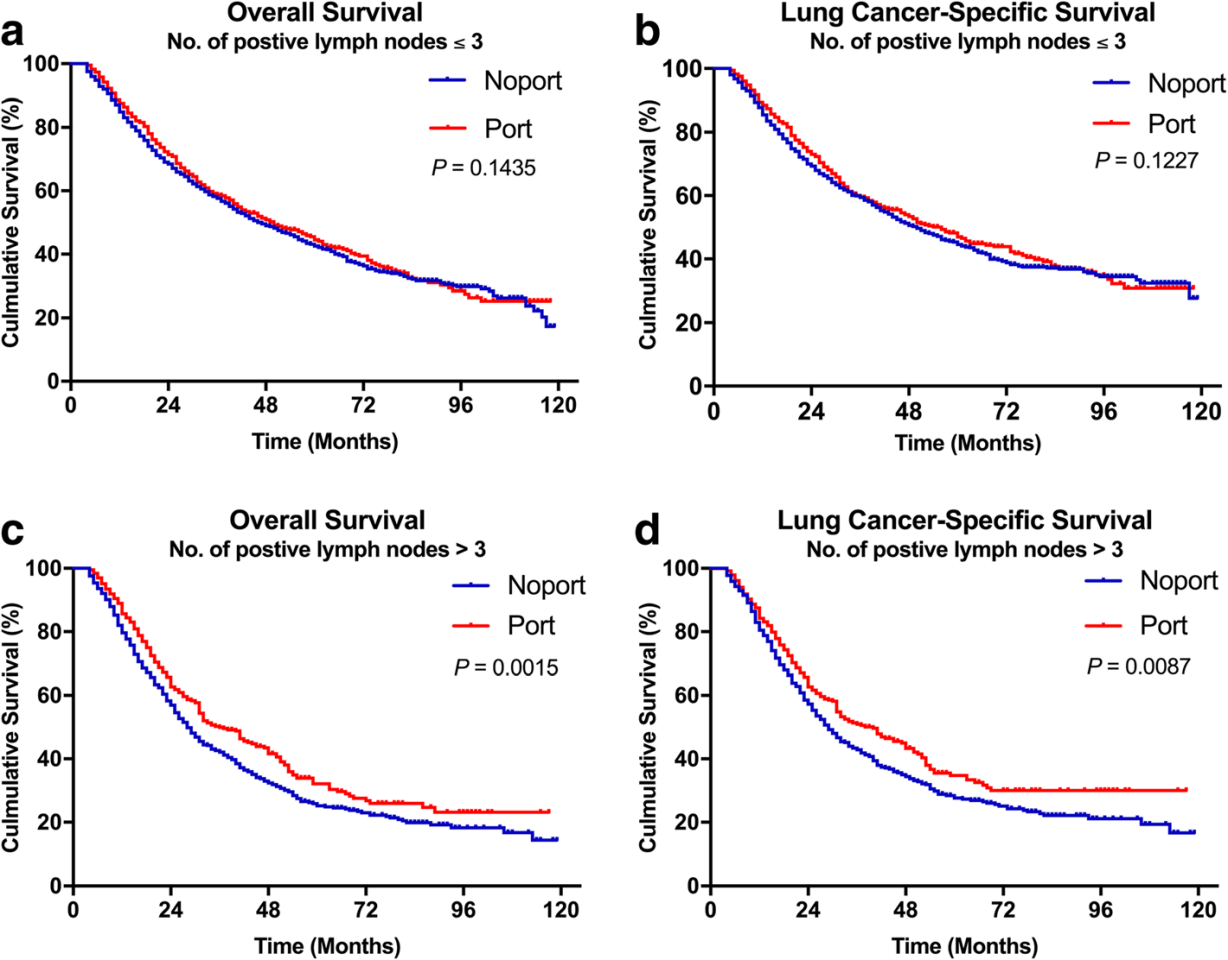
Overall and lung cancer-specific survivals in stage IIIA-pN2 patients with the exact number of positive lymph nodes ≤3 (**a** and **b**) or >3 (**c** and **d**) undergoing No PORT or PORT. Abbreviations: PORT, Postoperative radiation. NSCLC, Non-small cell lung cancer

The survival analyses were also investigated based on positive LNs categories (*n* ≤ 3 and *n* > 3). For patients with positive LNs (n ≤ 3), no significant differences was observed in OS (*p* = 0·1435) and LCSS (*p* = 0·1227) (Fig. 3a and b). However, for patients with positive LNs (n > 3), there was a significant difference in survival between PORT and No PORT both in OS (*p* = 0·0015) and LCSS (p = 0·0087) (Fig. 3 c and d).

The Cox proportional hazards regression model (Table 2) was then applied to study the superiority of PORT in subgroups of positive LNs categories (n ≤ 3 and n > 3). In the positive LNs (n ≤ 3) subgroup, the use of PORT did not have a significant impact on survival both in OS and LCSS (OS with No PORT vs. PORT: HR, 0·887; 95% CI, 0·778 to 1·011; p = 0·072; LCSS with No PORT vs. PORT: HR, 0·897; 95% CI, 0·774 to 1·033; p = 0·129). In positive LNs (n > 3), the use of PORT was associated with an improved statistical survival both in OS and LCSS (OS with No PORT vs. PORT: HR, 0·803; 95% CI, 0·687 to 0·938; p = 0·006; LCSS with No PORT vs. PORT: HR, 0·794; 95% CI, 0·671 to 0·94; p = 0·007).

**Table 2:** Cox Proportional Hazards Regression Model for Overall Survival and Lung Cancer-Specific Survival in Patients based on Number Categories

	No. of positive lymph nodes ≤3				No. of positive lymph nodes >3			
	Overall Survival		Lung Cancer-Specific Survival		Overall Survival		Lung Cancer-Specific Survival	
Variable	Hazard Ratio (95% CI)	*p*	Hazard Ratio (95% CI)	*p*	Hazard Ratio (95% CI)	*p*	Hazard Ratio (95% CI)	*p*
Age, years								
< 65	1.00 (reference)		1.00 (reference)		1.00 (reference)		1.00 (reference)	
≥ 65	1.449(1.279 to 1.643)	<0.001	1.469(1.281 to 1.684)	<0.001	1.345(1.152 to 1.572)	<0.001	1.381(1.168 to 1.632)	<0.001
Race		0.234		0.396		0.054		0.068
White	1.00 (reference)		1.00 (reference)		1.00 (reference)		1.00 (reference)	
Black	1.030(0.849 to 1.249)	0.768	1.012(0.817 to 1.255)	0.91	0.739(0.547 to 0.998)	0.048	0.757(0.557 to 1.031)	0.077
Other	0.832(0.668 to 1.037)	0.102	0.849(0.667 to 1.08))	0.182	0.802(0.606 to 1.061)	0.122	0.776(0.572 to 1.052)	0.103
Sex								
Female	1.00 (reference)		1.00 (reference)		1.00 (reference)		1.00 (reference)	
Male	1.349(1.191 to 1.528)	<0.001	1.366(1.192 to 1.567)	<0.001	1.308(1.122 to 1.525)	0.001	1.279(1.085 to 1.507)	0.003
Primary Site		0.705		0.963		0.993		0.976
Main bronchus	1.00 (reference)		1.00 (reference)		1.00 (reference)		1.00 (reference)	
Upper lobe	1.189(0.634 to 2.230)	0.59	0.986(0.493 to 1.969)	0.967	1.074(0.624 to 1.85)	0.796	1.18(0.656 to 2.121)	0.581
Middle lobe	1.384(0.699 to 2.739)	0.351	1.065(0.502 to 2.26)	0.87	0.988(0.491 to 1.988)	0.973	1.096(0.523 to 2.3)	0.808
Lower lobe	1.269(0.673 to 2.394)	0.462	1.035(0.514 to 2.084)	0.923	1.088(0.626 to 1.892)	0.765	1.195(0.658 to 2.172)	0.558
Overlapping/ lung, NOS	1.241(0.616 to 2.500)	0.545	1.021(0.474 to 2.198)	0.957	1.055(0.535 to 2.083)	0.876	1.124(0.548 to 2.306)	0.749
Histology		0.11		0.043		0.152		0.13
Non-small cell carcinoma	1.00 (reference)		1.00 (reference)		1.00 (reference)		1.00 (reference)	
Adenocarcinoma, NOS	0.888(0.701 to 1.125)	0.325	0.861(0.665 to 1.115)	0.257	1.129(0.836 to 1.525)	0.428	1.222(0.876 to 1.705)	0.237
Squamous cell carcinoma, NOS	1.121(0.874 to 1.437)	0.368	1.047(0.797 to 1.375)	0.741	1.248(0.905 to 1.722)	0.177	1.456(1.018 to 2.081)	0.039
Large cell carcinoma, NOS	1.12(0.765 to 1.64)	0.561	1.16(0.779 to 1.729)	0.465	1.778(0.867 to 3.122)	0.045	1.536(0.795 to 2.966)	0.201
Laterality								
Left	1.00 (reference)		1.00 (reference)		1.00 (reference)		1.00 (reference)	
Right	0.985(0.871 to 1.114)	0.809	1.008(0.88 to 1.154)	0.911	1.012(0.867 to 1.18)	0.882	1.05(0.889 to 1.239)	0.568
Tumor Size		0.66		0.724		0.463		0.5
≤ 3.0	1.00 (reference)		1.00 (reference)		1.00 (reference)		1.00 (reference)	
3.1-5.0	1.008(0.838 to 1.213)	0.93	1.045(0.854 to 1.385)	0.671	1.068(0.849 to 1.343)	0.575	1.061(0.827 to 1.36)	0.641
5.1-7.0	1.03(0.825 to 1.286)	0.793	1.088(0.854 to 1.385)	0.495	1.146(0.879 to 1.496)	0.314	1.13(0.85 to 1.503)	0.4
≥ 7.1	0.879(0.661 to 1.168)	0.374	0.936(0.684 to 1.28)	0.678	1.216(0.888 to 1.665)	0.222	1.256(0.892 to 1.767)	0.192
Unknown	1.297(0.766 to 2.196)	0.332	1.344(0.765 to 2.362)	0.304	0.66(0.302 to 1.44)	0.296	0.692(0.316 to 1.515)	0.357
T stage		0.001		0.001		0.003		0.005
T1	1.00 (reference)		1.00 (reference)		1.00 (reference)		1.00 (reference)	
T2	1.296(1.072 to 1.566)	0.007	1.279(1.037 to 1.578)	0.022	1.207(0.933 to 1.562)	0.151	1.223(0.925 to 1.616)	0.157
T3	1.722(1.324 to 2.239)	<0.001	1.79(1.348 to 2.375)	<0.001	1.769(1.291 to 2.423)	<0.001	1.793(1.279 to 2.514)	0.001
Tx	1.028(0.404 to 2.614)	0.954	1.11(0.43 to 2.87)	0.829	1.172(0.312 to 4.412)	0.814	1.242(0.329 to 4.697)	0.749
No. of positive lymph code^a^		0.389		0.17		0.074		0.075
1/4	1.00 (reference)		1.00 (reference)		1.00 (reference)		1.00 (reference)	
2/5	1.038(0.902 to 1.194)	0.601	1.032(0.884 to 1.205)	0.689	1.288(1.035 to 1.604)	0.024	1.316(1.039 to 1.668)	0.023
3/6	1.113(0.955 to 1.297)	0.169	1.172(0.991 to 1.387)	0.064	1.197(0.937 to 1.528)	0.15	1.24(0.952 to 1.616)	0.111
≥ 7					1.255(1.036 to 1.52)	0.02	1.275(1.036 to 1.569)	0.022
Surgery type								
Lobectomy	1.00 (reference)		1.00 (reference)		1.00 (reference)		1.00 (reference)	
Pneumonectomy	1.101(0.884 to 1.372)	0.391	1.056(0.822 to 1.358)	0.669	0.986(0.791 to 1.23)	0.903	1.025(0.812 to 1.293)	0.836
Port								
No	1.00 (reference)		1.00 (reference)		1.00 (reference)		1.00 (reference)	
Yes	0.887(0.778 to 1.011)	0.072	0.894(0.774 to 1.033)	0.129	0.803(0.687 to 0.938)	0.006	0.794(0.671 to 0.94)	0.007

*Abbreviations*: *NOS* not otherwise specified

^a^: 4, 5, 6, ≥7, these four categoric variables were designed for hazard ratios in the right two columns

## Discussion

Through a large population-based cohort based on SEER database, we investigated whether the use of PORT will improve the prognosis of patients examined rare LNs metastasis. We detected age, race, sex, primary site, histology and so on when analyzing both OS and LCSS in pooled analysis. The outcomes of multivariable analyses demonstrated that the number of positive LNs and the use of PORT were independent risk factors. Patients with positive lymph more than 3 were found with poorer survival, and the use of PORT benefited patients significantly. These independent risk results were similar to a previous SEER based study and a retrospective study [4, 11]. In order to detect how the number of positive LNs influences the OS and LCSS after the use of PORT, we assigned patients into positive LNs (*n* ≤ 3) group and positive LNs (*n* > 3) group according to the hazard ratios of each number category. Consequently, Kaplan-Meier curves and the cox proportional hazards regression models all demonstrated that the use of PORT significant improves survival for the patients with positive LNs (n > 3). And PORT was found not associated with the survival benefit in patients with positive LNs (n ≤ 3).

The SEER data are retrospectively collected, so the potential for error or bias may exist. We recognize that confounding factors, such as margin status and performance status, may influence the treating physician’s decision to recommend the use of PORT. This information is not available for analysis, and an estimated 1% to 17% of surgical resections could still result in positive surgical margins [12]. So we only selected patients underwent lobectomy or pneumonectomy to avoid the positive margin status bias as much as possible. In addition, patients underwent lobectomy or pneumonectomy tend to have a better performance status than those who taken partial, wedge or segmental resection. Details about the lymph resection are also not available in the database. According to NCCN guidelines, resection is considered not appropriate for patients with multiple pathologically proven malignant LNs greater than 3 cm, so those patients with fusion or huge malignant LNs could have been be excluded after patient selection. Considering more than 3000 patients were included in this study, we hoped that the impact of the incomplete LNs resection could be minimized.

To date, SEER database does not provide the data of adjuvant chemotherapy or target therapy, and SEER-Medicare database does not open to the users outside the United States. Although preoperative adjuvant chemotherapy is now considered the standard treatment for resectable pN2 status patients, more detail data is still need for the further research. For patients with PORT, details of the dose, range and the dose per fraction are not available as well. Presently, the standard PORT dose and dose per fraction were considered less than 54 Gy and 2 Gy respectively [6, 13]. According to the latest NCCN guide lines, the total dose of PORT was recommended no more than 60 Gy and the dose per faction was recommended less than 2Gy. 50-54Gy was recommended for negative margins and 54-60Gy was recommended for extracapsular nodal extension or microscopic positive margins in guide lines. According to a previous SEER and National Cancer Data Base (NCDB) pooled analysis, NCDB contains data not available in SEER database, such as chemotherapy and RT dose. And the results demonstrated that the range of radiotherapy dose is from 45 to 82.8 Gy and the median dose is 54 Gy after screening patients [14]. Therefore, we only included patients underwent lobectomy or pneumonectomy to reduce the heterogeneity of radiotherapy stems from positive margins and different performance status.

## Conclusions

In summary, results from our study demonstrated that the exact number of positive LNs in ipsilateral mediastinal nodal resection has an impact on survival for stage IIIA pN2 patients with PORT. Although there were biases in lacking the details of adjuvant chemotherapy and postoperative radiotherapy, standard chemotherapy regiments and modern radiation technology could minimize these biases. To our knowledge, PORT was deemed detrimental for patients with N0 or N1 nodal disease because of the increased rate of intercurrent deaths [4, 15, 16]. One explanation is undetected microscopic/residual is less in N0 and N1 disease, so the benefit gained by treatment with PORT is diminished from the radiation toxicity [4]. For pN2 nodal status, there is a larger lymphatic metastasis of disease compared with N0 or N1, so the use of PORT is often recommended. According to our results, different number of positive LNs seem to have an impact on the survival benefit from the use of PORT. The results of LCSS analysis suggested a lower rate of recurrence for patients with more than 3 positive LNs, which could be considered the benefit from the PORT defeated the toxicity. All in all, we concluded that the number category is a strong independent prognostic factor in NSCLC and could add new information to the use of PORT in NSCLC pN2 status patients. Meanwhile, our results support the need to enroll patients on randomized controlled trials for the further analysis.

## Additional file

Additional file 1:
**Table S1.** Cox proportional hazards regression model for overall survival and lung cancer-specific survival in patients with stage IIIA pN2 status NSCLC. Abbreviations: NSCLC, Non-small cell lung cancer. NOS, Not Otherwise Specified. **Table S2.** Cox proportional hazards regression model for overall survival and lung cancer-specific survival in patients with stage IIIA pN2 status NSCLC. (No. of positive lymph nodes in two categories). Abbreviations: NSCLC, Non-small cell lung cancer. NOS, Not Otherwise Specified. **Table S3.** Baseline characteristics of Patients with NSCLC in overall survival analysis. *: 4, 5, 6, ≥7, these four categoric variables were designed for the right two columns. Abbreviations: NSCLC, Non-small cell lung cancer. NOS, Not Otherwise Specified. **Table S4.** Baseline characteristics of Patients with NSCLC in lung cancer-specific survival analysis. *: 4, 5, 6, ≥7, these four categoric variables were designed for the right two columns. Abbreviations: NSCLC, Non-small cell lung cancer. NOS, Not Otherwise Specified. (DOCX 68 kb)

## Abbreviations

AJCC: American Association of Cancer
HRs: Hazard ratios
ICD-O-3: International Classification of Diseases for Oncology, 3rd Edition
LCSS: Lung cancer-specific survival
LNs: Lymph nodes
LRR: Localregional recurrence
NCCN: National Comprehensive Cancer Network
NCDB: National Cancer Data Base
NOS: Not otherwise specified
NSCLC: Non-small cell lung cancer
OS: Overall survival
pN2: Pathological N2 status
PORT: Postoperative radiation
SEER: Surveillance, epidemiology, and end results
WHO: World Health Organization
