# Supplementary material for: Choice of postoperative radiation for stage IIIA pathologic N2 non-small cell lung cancer: impact of metastatic lymph node number
Source: Radiat Oncol. 2017 Dec 29;12:207. doi: 10.1186/s13014-017-0946-1 (PMC5747172; doi:10.1186/s13014-017-0946-1)
Supplement: Additional file 1: — Table S1. Cox proportional hazards regression model for overall survival and lung cancer-specific survival in patients with stage IIIA pN2 status NSCLC. Abbreviations: NSCLC, Non-small cell lung cancer. NOS, Not Otherwise Specified. Table S2. Cox proportional hazards regression model for overall survival and lung cancer-specific survival in patients with stage IIIA pN2 status NSCLC. (No. of positive lymph nodes in two categories). Abbreviations: NSCLC, Non-small cell lung cancer. NOS, Not Otherwise Specified. Table S3. Baseline characteristics of Patients with NSCLC in overall survival analysis. *: 4, 5, 6, ≥7, these four categoric variables were designed for the right two columns. Abbreviations: NSCLC, Non-small cell lung cancer. NOS, Not Otherwise Specified. Table S4. Baseline characteristics of Patients with NSCLC in lung cancer-specific survival analysis. *: 4, 5, 6, ≥7, these four categoric variables were designed for the right two columns. Abbreviations: NSCLC, Non-small cell lung cancer. NOS, Not Otherwise Specified. (DOCX 68 kb) [file 13014_2017_946_MOESM1_ESM.docx]

| **Additional file 1:**  Table S1: Cox proportional hazards regression model for overall survival and lung cancer-specific survival in patients with stage IIIA pN2 status NSCLC.  Abbreviations: NSCLC, Non-small cell lung cancer. NOS, Not Otherwise Specified.  Table S2: Cox proportional hazards regression model for overall survival and lung cancer-specific survival in patients with stage IIIA pN2 status NSCLC. (No. of positive lymph nodes in two categories)  Abbreviations: NSCLC, Non-small cell lung cancer. NOS, Not Otherwise Specified.  Table S3: Baseline characteristics of Patients with NSCLC in overall survival analysis.  *: 4, 5, 6, ≥7, these four categoric variables were designed for the right two columns.  Abbreviations: NSCLC, Non-small cell lung cancer. NOS, Not Otherwise Specified.  Table S4: Baseline characteristics of Patients with NSCLC in lung cancer-specific survival analysis.  *: 4, 5, 6, ≥7, these four categoric variables were designed for the right two columns.  Abbreviations: NSCLC, Non-small cell lung cancer. NOS, Not Otherwise Specified.  **Table S1: Cox Proportional Hazards Regression Model for Overall Survival and Lung Cancer-Specific Survival in Patients with stage IIIA pN2 status NSCLC** | | | | | |
| --- | --- | --- | --- | --- | --- |
|  | **Overall Survival** | |  | **Lung Cancer-Specific survival** | |
| **Variable** | Hazard Ratio (95% CI) | *p* |  | Hazard Ratio (95% CI) | *p* |
| **Age, years** |  |  |  |  |  |
| <65 | 1.00 (reference) |  |  | 1.00 (reference) |  |
| ≥65 | 1.414(1.287 to 1.553) | <0.001 |  | 1.438(1.299 to 1.592) | <0.001 |
| **Race** |  | 0.059 |  |  | 0.066 |
| White | 1.00 (reference) |  |  | 1.00 (reference) |  |
| Black | 0.916(0.781 to 1.075) | 0.282 |  | 0.903(0.759 to 1.074) | 0.248 |
| Other | 0.827(0.699 to 0.978) | 0.027 |  | 0.819(0.682 to 0.984) | 0.033 |
| **Sex** |  |  |  |  |  |
| Female | 1.00 (reference) |  |  | 1.00 (reference) |  |
| Male | 1.32(1.202 to 1.449) | <0.001 |  | 1.315(1.188 to 1.455) | <0.001 |
| **Primary Site** |  | 0.836 |  |  | 0.948 |
| Main bronchus | 1.00 (reference) |  |  | 1.00 (reference) |  |
| Upper lobe | 1.083(0.725 to 1.619) | 0.696 |  | 1.04(0.673 to 1.609) | 0.859 |
| Middle lobe | 1.187(0.753 to 1.872) | 0.459 |  | 1.098(0.67 to 1.8) | 0.71 |
| Lower lobe | 1.123(0.748 to 1.686) | 0.575 |  | 1.074(0.691 to 1.671) | 0.75 |
| Overlapping/ lung, NOS | 1.025(0.644 to 1.631) | 0.918 |  | 0.985(0.597 to 1.627) | 0.954 |
| **Histology** |  | 0.006 |  |  | 0.018 |
| Non-small cell carcinoma | 1.00 (reference) |  |  | 1.00 (reference) |  |
| Adenocarcinoma, NOS | 0.947(0.794 to 1.13) | 0.546 |  | 0.947(0.781 to 1.148) | 0.581 |
| Squamous cell carcinoma, NOS | 1.133(0.939 to 1.366) | 0.192 |  | 1.13(0.92 to 1.388) | 0.243 |
| Large cell carcinoma, NOS | 1.21(0.894 to 1.637) | 0.217 |  | 1.191(0.861 to 1.647) | 0.292 |
| **Laterality** |  |  |  |  |  |
| Left | 1.00 (reference) |  |  | 1.00 (reference) |  |
| Right | 0.995(0.907 to 1.092) | 0.913 |  | 1.022(0.923 to 1.131) | 0.678 |
| **Tumor Size** |  | 0.703 |  |  | 0.617 |
| ≤3.0 | 1.00 (reference) |  |  | 1.00 (reference) |  |
| 3.1-5.0 | 1.048(0.912 to 1.205) | 0.511 |  | 1.067(0.917 to 1.242) | 0.401 |
| 5.1-7.0 | 1.109(0.941 to 1.306) | 0.217 |  | 1.139(0.954 to 1.359) | 0.15 |
| ≥7.1 | 1.026(0.837 to 1.258) | 0.804 |  | 1.063(0.851 to 1.329) | 0.589 |
| Unknown | 0.908(0.616 to 1.34) | 0.628 |  | 0.917(0.608 to 1.383) | 0.68 |
| **T stage** |  | <0.001 |  |  | <0.001 |
| T1 | 1.00 (reference) |  |  | 1.00 (reference) |  |
| T2 | 1.24(1.07 to 1.437) | 0.004 |  | 1.233(1.048 to 1.449) | 0.011 |
| T3 | 1.736(1.432 to 2.104) | <0.001 |  | 1.772(1.44 to 2.181) | <0.001 |
| Tx | 1.239(0.665 to 2.308) | 0.499 |  | 1.347(0.715 to 2.538) | 0.356 |
| **No. of positive lymph code** |  | <0.001 |  |  | <0.001 |
| 1 | 1.00 (reference) |  |  | 1.00 (reference) |  |
| 2 | 1.027(0.893 to 1.18) | 0.712 |  | 1.02(0.875 to 1.189) | 0.797 |
| 3 | 1.096(0.942 to 1.275) | 0.236 |  | 1.15(0.974 to 1.357) | 0.1 |
| 4 | 1.202(1.014 to 1.426) | 0.034 |  | 1.232(1.023 to 1.485) | 0.028 |
| 5 | 1.553(1.29 to 1.868) | <0.001 |  | 1.61(1.319 to 1.965) | <0.001 |
| 6 | 1.473(1.188 to 1.825) | <0.001 |  | 1.56(1.236 to 1.968) | <0.001 |
| 7 | 1.313(1.006 to 1.714) | 0.045 |  | 1.364(1.013 to 1.836) | 0.041 |
| ≥8 | 1.591(1.352 to 1.873) | <0.001 |  | 1.626(1.364 to 1.973) | <0.001 |
| No. of unspecified | 1.224(0.998 to 1.501) | 0.052 |  | 1.264(1.015 to 1.573) | 0.036 |
| **Surgery type** |  |  |  |  |  |
| Lobectomy | 1.00 (reference) |  |  | 1.00 (reference) |  |
| Pneumonectomy | 1.061(0.914 to 1.232) | 0.434 |  | 1.065(0.904 to 1.253) | 0.451 |
| **Port** |  |  |  |  |  |
| No | 1.00 (reference) |  |  | 1.00 (reference) |  |
| Yes | 0.854(0.776 to 0.941) | 0.001 |  | 0.855(0.769 to 0.95) | 0.004 |

| **Table S2: Cox Proportional Hazards Regression Model for Overall Survival and Lung Cancer-Specific Survival in Patients with stage IIIA pN2 status NSCLC (No. of positive lymph nodes in two categories)** | | | | | |
| --- | --- | --- | --- | --- | --- |
|  | **Overall Survival** | |  | **Lung Cancer-Specific survival** | |
| **Variable** | Hazard Ratio (95% CI) | *p* |  | Hazard Ratio (95% CI) | *p* |
| **Age, years** |  |  |  |  |  |
| <65 | 1.00 (reference) |  |  | 1.00 (reference) |  |
| ≥65 | 1.407 (1.281 to 1.545) | <0.001 |  | 1.428 (1.290 to 1.581) | <0.001 |
| **Race** |  | 0.055 |  |  | 0.055 |
| White | 1.00 (reference) |  |  | 1.00 (reference) |  |
| Black | 0.909 (0.776 to 1.066) | 0.242 |  | 0.895 (0.753 to 1.063) | 0.207 |
| Other | 0.828 (0.699 to 0.979) | 0.027 |  | 0.817 (0.680 to 0.982) | 0.031 |
| **Sex** |  |  |  |  |  |
| Female | 1.00 (reference) |  |  | 1.00 (reference) |  |
| Male | 1.321 (1.204 to 1.450) | <0.001 |  | 1.316 (1.189 to 1.455) | <0.001 |
| **Primary Site** |  | 0.768 |  |  | 0.901 |
| Main bronchus | 1.00 (reference) |  |  | 1.00 (reference) |  |
| Upper lobe | 1.112 (0.745 to 1.660) | 0.603 |  | 1.079 (0.699 to 1.666) | 0.73 |
| Middle lobe | 1.223 (0.776 to 1.926) | 0.386 |  | 1.142 (0.698 to 1.869) | 0.596 |
| Lower lobe | 1.163 (0.776 to 1.743) | 0.465 |  | 1.126 (0.726 to 1.747) | 0.597 |
| Overlapping/ lung, NOS | 1.061 (0.667 to 1.686) | 0.803 |  | 1.029 (0.625 to 1.695) | 0.911 |
| **Histology** |  | 0.009 |  |  | 0.026 |
| Non-small cell carcinoma | 1.00 (reference) |  |  | 1.00 (reference) |  |
| Adenocarcinoma, NOS | 0.947 (0.794 to 1.129) | 0.545 |  | 0.951 (0.785 to 1.152) | 0.607 |
| Squamous cell carcinoma, NOS | 1.123 (0.931 to 1.354) | 0.225 |  | 1.125 (0.917 to 1.381) | 0.26 |
| Large cell carcinoma, NOS | 1.205 (0.891 to 1.630) | 0.227 |  | 1.187 (0.859 to 1.642) | 0.299 |
| **Laterality** |  |  |  |  |  |
| Left | 1.00 (reference) |  |  | 1.00 (reference) |  |
| Right | 0.994 (0.906 to 1.091) | 0.898 |  | 1.020 (0.922 to 1.129) | 0.699 |
| **Tumor Size** |  | 0.666 |  |  | 0.553 |
| ≤3.0 | 1.00 (reference) |  |  | 1.00 (reference) |  |
| 3.1-5.0 | 1.045 (0.909 to 1.201) | 0.535 |  | 1.064 (0.914 to 1.238) | 0.422 |
| 5.1-7.0 | 1.115 (0.947 to 1.313) | 0.193 |  | 1.149 (0.963 to 1.371) | 0.124 |
| ≥7.1 | 1.032 (0.842 to 1.265) | 0.759 |  | 1.065 (0.852 to 1.331) | 0.582 |
| Unknown | 0.906 (0.615 to 1.336) | 0.619 |  | 0.912 (0.605 to 1.374) | 0.66 |
| **T stage** |  | <0.001 |  |  | <0.001 |
| T1 | 1.00 (reference) |  |  | 1.00 (reference) |  |
| T2 | 1.254 (1.082 to 1.453) | 0.003 |  | 1.247 (1.061 to 1.466) | 0.007 |
| T3 | 1.770 (1.461 to 2.144) | <0.001 |  | 1.810 (1.472 to 2.225) | <0.001 |
| Tx | 1.229 (0.660 to 2.287) | 0.516 |  | 1.340 (0..712 to 2.524) | 0.364 |
| **No. of positive lymph code** |  | <0.001 |  |  | <0.001 |
| ≤3 | 1.00 (reference) |  |  | 1.00 (reference) |  |
| >3 | 1.379 (1.253 to 1.519) | <0.001 |  | 1.415 (1.274 to 1.571) | <0.001 |
| Unspecified | 1.189 (0.980 to 1.443) | 0.08 |  | 1.217 (0.989 to 1.497) | 0.063 |
| **Surgery type** |  |  |  |  |  |
| Lobectomy | 1.00 (reference) |  |  | 1.00 (reference) |  |
| Pneumonectomy | 1.069 (0.921 to 1.240) | 0.382 |  | 1.075 (0.914 to 1.265) | 0.384 |
| **Port** |  |  |  |  |  |
| No | 1.00 (reference) |  |  | 1.00 (reference) |  |
| Yes | 0.860 (0.781 to 0.947) | 0.002 |  | 0.862 (0.775 to 0.957) | 0.006 |

| **Table S3: Baseline Characteristics of Patients With NSCLC in Overall Survival analysis** | | | | | | | |
| --- | --- | --- | --- | --- | --- | --- | --- |
|  | **No. of Patients by number of positive lymph nodes (number unspecified excluded)** | | | | | | |
|  | **≤3 (n = 2030)** | |  |  | **>3 (n = 1153)** | |  |
| **Demographic** | No Port/1359 | Port/671 | P Value for χ2 |  | No Port/696 | Port/457 | P Value for χ2 |
| **Age at diagnosis, years** |  |  |  |  |  |  |  |
| <65 | 551（40.5%） | 343(51.1%) | <0.001 |  | 278(39.9%) | 239(52.3%) | <0.001 |
| ≥65 | 808(59.5%) | 328(48.9%) |  |  | 418(60.1%) | 218(47.7%) |  |
| **Sex** |  |  |  |  |  |  |  |
| Male | 690(50.8%) | 342(51.0%) | 0.934 |  | 363(52.2%) | 231(50.5%) | 0.593 |
| Female | 669(49.2%) | 329(49.0%) |  |  | 333(47.8%) | 226(49.5%) |  |
| **Race** |  |  |  |  |  |  |  |
| White | 1096(80.6%) | 528(78.7%) | 0.525 |  | 578(83.0%) | 376(82.3%) | 0.291 |
| Black | 141(10.4%) | 80(11.9%) |  |  | 64(9.2%) | 35(7.6%) |  |
| Other | 122(9.0%) | 63(9.4%) |  |  | 54(7.8%) | 46(10.1%) |  |
| **Location** |  |  |  |  |  |  |  |
| Main bronchus | 13(1.0%) | 6(0.9%) | 0.069 |  | 14(2.0%) | 8(1.8%) | 0.72 |
| Upper lobe | 757(55.7%) | 416(62.0%) |  |  | 393(56.5%) | 267(58.4%) |  |
| Middle lobe | 71(5.2%) | 35(5.2%) |  |  | 24(3.4%) | 16(3.5%) |  |
| Lower lobe | 480(35.3%) | 194(28.9%) |  |  | 240(34.5%) | 156(34.1%) |  |
| Overlapping/lung, NOS | 38(2.8%) | 20(3.0%) |  |  | 25(3.6%) | 10(2.2%) |  |
| **T stage** |  |  |  |  |  |  |  |
| T1 | 414(30.4%) | 204(30.4%) | 0.014 |  | 160(23.0%) | 105(23.0%) | 0.909 |
| T2 | 800(58.9%) | 365(54.4%) |  |  | 445(63.9%) | 296(64.8%) |  |
| T3 | 136(10.0%) | 99(14.8%) |  |  | 88(12.7%) | 53(11.6%) |  |
| TX | 9(0.7%) | 3(0.4%) |  |  | 3(0.4%) | 3(0.6%) |  |
| **Tumor size, cm** |  |  |  |  |  |  |  |
| ≤3.0 | 620(45.6%) | 301(44.9%) | 0.609 |  | 267(38.4%) | 178(39.0%) | 0.908 |
| 3.1 to 5.0 | 440(32.4%) | 213(31.7%) |  |  | 241(34.6%) | 165(36.1%) |  |
| 5.1 to 7.0 | 186(13.7%) | 88(13.1%) |  |  | 121(17.4%) | 70(15.3%) |  |
| ≥7.1 | 92(6.8%) | 59(8.8%) |  |  | 57(8.2%) | 37(8.1%) |  |
| Unknown | 21(1.5%) | 10(1.5%) |  |  | 10(1.4%) | 7(1.5%) |  |
| **Laterality** |  |  |  |  |  |  |  |
| Right | 758(55.8%) | 391(58.4%) | 0.27 |  | 368(52.9%) | 254(55.7%) | 0.346 |
| Left | 601(44.2%) | 279(41.6%) |  |  | 328(47.1%) | 202(44.3%) |  |
| Unspecified | / | 1 |  |  | / | 1 |  |
| **Histology** |  |  |  |  |  |  |  |
| Non-small cell carcinoma | 84(6.2%) | 46(6.9%) | 0.479 |  | 47(6.8%) | 28(6.1%) | 0.415 |
| Adenocarcinoma, NOS | 833(61.3%) | 429(63.9%) |  |  | 458(65.8%) | 322(70.5%) |  |
| Squamous cell carcinoma, NOS | 398(29.3%) | 178(26.5%) |  |  | 175(25.1%) | 99(21.7%) |  |
| Large cell carcinoma, NOS | 44(3.2%) | 18(2.7%) |  |  | 16(2.3%) | 8(1.7%) |  |
| **Surgery type** |  |  |  |  |  |  |  |
| Lobectomy | 1219(89.7%) | 628(93.6%) | 0.004 |  | 566(81.3%) | 396(86.7%) | 0.017 |
| Pneumonectomy | 140(10.3%) | 43(6.4%) |  |  | 130(18.7%) | 61(13.3%) |  |
| **Positive lymph nodes*** |  |  |  |  |  |  |  |
| 1/4 | 655 (48.2%) | 286(42.6%) | 0.013 |  | 198(28.4%) | 116(25.4%) | 0.4 |
| 2/5 | 426 (31.3%) | 212(31.6%) |  |  | 135(19.4%) | 103(22.5%) |  |
| 3/6 | 278 (20.5%) | 173(25.8%) |  |  | 101(14.5%) | 59(12.9%) |  |
| ≥7 |  |  |  |  | 262(37.7%) | 179(39.2%) |  |

| **Table S4: Baseline Characteristics of Patients With NSCLC in Lung Cancer-Specific analysis** | | | | | | | |
| --- | --- | --- | --- | --- | --- | --- | --- |
|  | **No. of Patients by number of positive lymph nodes (number unspecified excluded)** | | | | | | |
|  | **≤3 (n = 1840)** | |  |  | **>3 (n = 1057)** | |  |
| **Demographic** | No Port/1227 | Port/613 | P Value for χ2 |  | No Port/642 | Port/415 | P Value for χ2 |
| **Age at diagnosis, years** |  |  |  |  |  |  |  |
| <65 | 515(42.0%) | 318(51.9%) | <0.001 |  | 260(40.4%) | 221(53.3%) | <0.001 |
| ≥65 | 712(58.0%) | 295(48.1%) |  |  | 382(59.5%) | 194(46.7%) |  |
| **Sex** |  |  |  |  |  |  |  |
| Male | 618(50.4%) | 304(49.6%) | 0.754 |  | 329(51.2%) | 207(49.9%) | 0.664 |
| Female | 609(49.6%) | 309(50.4%) |  |  | 313(48.8%) | 208(50.1%) |  |
| **Race** |  |  |  |  |  |  |  |
| White | 989(80.6%) | 483(78.8%) | 0.64 |  | 529(82.4%) | 340(81.9%) | 0.366 |
| Black | 127(10.4%) | 71(11.6%) |  |  | 63(9.8%) | 34(8.2%) |  |
| Other | 111(9.0%) | 59(9.6%) |  |  | 50(7.8%) | 41(9.9%) |  |
| **Location** |  |  |  |  |  |  |  |
| Main bronchus | 13(1.1%) | 4(0.7%) | 0.07 |  | 13(2.0%) | 7(1.7%) | 0.798 |
| Upper lobe | 691(56.3%) | 384(62.6%) |  |  | 361(56.3%) | 236(56.9%) |  |
| Middle lobe | 62(5.1%) | 32(5.2%) |  |  | 24(3.7%) | 15(3.6%) |  |
| Lower lobe | 426(34.7%) | 174(28.4%) |  |  | 220(34.3%) | 147(35.4%) |  |
| Overlapping/lung, NOS | 35(2.8%) | 19(3.1%) |  |  | 24(3.7%) | 10(2.4%) |  |
| **T stage** |  |  |  |  |  |  |  |
| T1 | 364(29.7%) | 189(30.8%) | 0.027 |  | 152(23.7%) | 93(22.4%) | 0.637 |
| T2 | 724(59.0%) | 329(53.7%) |  |  | 401(62.4%) | 272(65.6%) |  |
| T3 | 130(10.6%) | 92(15.0%) |  |  | 86(13.4%) | 47(11.3%) |  |
| TX | 9(0.7%) | 3(0.5%) |  |  | 3(0.5%) | 3(0.7%) |  |
| **Tumor size, cm** |  |  |  |  |  |  |  |
| ≤3.0 | 548(44.7%) | 281(45.8%) | 0.459 |  | 253(39.4%) | 155(37.3%) | 0.619 |
| 3.1 to 5.0 | 405(33.0%) | 191(31.2%) |  |  | 213(33.2%) | 157(37.8%) |  |
| 5.1 to 7.0 | 172(14.0%) | 78(12.7%) |  |  | 114(17.8%) | 65(15.7%) |  |
| ≥7.1 | 82(6.7%) | 54(8.8%) |  |  | 52(8.1%) | 31(7.5%) |  |
| Unknown | 20(1.6%) | 9(1.5%) |  |  | 10(1.5%) | 7(1.7%) |  |
| **Laterality** |  |  |  |  |  |  |  |
| Right | 688(56.1%) | 368(60.1%) | 0.097 |  | 339(52.8%) | 232(56.0%) | 0.303 |
| Left | 539(43.9%) | 244(39.9%) |  |  | 303(47.2%) | 182(44.0%) |  |
| Unspecified | / | 1 |  |  | / | 1 |  |
| **Histology** |  |  |  |  |  |  |  |
| Non-small cell carcinoma | 75(6.1%) | 42(6.9%) | 0.337 |  | 44(6.8%) | 22(5.3%) | 0.369 |
| Adenocarcinoma, NOS | 755(61.5%) | 398(64.9%) |  |  | 428(66.7%) | 297(71.6%) |  |
| Squamous cell carcinoma, NOS | 354(28.9%) | 155(25.3%) |  |  | 157(24.5%) | 90(21.7%) |  |
| Large cell carcinoma, NOS | 43(3.5%) | 18(2.9%) |  |  | 13(2.0%) | 6(1.4%) |  |
| **Surgery type** |  |  |  |  |  |  |  |
| Lobectomy | 1101(89.7%) | 580(94.6%) | <0.001 |  | 519(80.8%) | 357(86.0%) | 0.029 |
| Pneumonectomy | 126(10.3%) | 33(5.4%) |  |  | 123(19.2%) | 58(14.0%) |  |
| **Positive lymph nodes*** |  |  |  |  |  |  |  |
| 1/4 | 588(47.9%) | 263(42.9%) | 0.038 |  | 183(28.5%) | 103(24.8%) | 0.329 |
| 2/5 | 386(31.5%) | 194(31.6%) |  |  | 124(19.3%) | 96(23.1%) |  |
| 3/6 | 253(20.6%) | 156(25.5%) |  |  | 92(14.3%) | 54(13.0%) |  |
| ≥7 |  |  |  |  | 243(37.9%) | 162(39.1%) |  |
